# Inheritance of Resistance to Chickpea Fusarium Wilt Disease (*Fusarium oxysporum* f. sp. *ciceris* Race 2) in a Wide-Cross *Cicer arietinum* × *Cicer reticulatum* Mapping Family

**DOI:** 10.3390/genes15060819

**Published:** 2024-06-20

**Authors:** Abdulkarim Lakmes, Abdullah Jhar, Ari Sadanandom, Adrian Christopher Brennan, Abdullah Kahriman

**Affiliations:** 1 Department of Field Crops, Harran University, Sanliurfa 63100, Türkiye; abdulkarimlakmes1982@gmail.com (A.L.); abdjhar@gmail.com (A.J.); kahraman@harran.edu.tr (A.K.); 2 Biosciences Department, Durham University, Durham DH1 3LE, UK; ari.sadanandom@durham.ac.uk

**Keywords:** crop wild relative, disease phenotyping, legume, monogenic inheritance, plant pathogen, quantitative trait locus, wilt resistance

## Abstract

Chickpea (*Cicer arietinum*) is a major food legume providing high quality nutrition, especially in developing regions. Chickpea wilt (*Fusarium oxysporum* f. sp. *ciceris*) causes significant annual losses. Integrated disease management of Fusarium wilt is supported by resistant varieties. Relatively few resistance genes are known so there is value in exploring genetic resources in chickpea wild relatives. This study investigates the inheritance of Fusarium wilt resistance (race 2) in recombinant inbred lines (RILs) from a cross between a cultivated susceptible chickpea variety (Gokce) and a wild resistant *Cicer reticulatum* line (Kayat-077). RILs, parents, resistant and susceptible tester lines were twice grown in the greenhouse with inoculation and disease symptoms scored. DNA was extracted from dried leaves and individuals were single nucleotide polymorphism (SNP) genotyped. SNPs were placed on the reference chickpea genome and quantitative trait locus (QTL) mapping was performed. Significant QTL regions were examined using PulseDB to identify candidate genes. The results showed the segregation of Fusarium wilt resistance conforming to a single gene inheritance. One significant QTL was found at the start of chromosome 8, containing 138 genes, three of which were disease-resistance candidates for chickpea breeding.

## 1. Introduction

Chickpea (*C. arietinum* L.) is an ancient crop that was grown from about 7000 B.C. in the Middle East after having been domesticated from the wild species *C. reticulatum* (Ladizinsky) [1]. Chickpea is cultivated globally under varied environmental conditions with the main producer being India which produces over 65%, followed by Australia, producing 14% of the total global production in 2016 [2]. Thus, chickpea is an important part of the diet in both the developed and developing world. Chickpea is the third most important pulse crop in the world and is a highly nutritious source of protein, carbohydrates, fiber, vitamins, minerals, and other micronutrients [3,4,5,6]. As a consequence, chickpea is an important part of subsistence diets and food security, in particular for reducing childhood malnutrition [7].

Chickpea wilt caused by *F. oxysporum* (Schlechtend.:Fr.) f. sp. *ciceris* (Padwick) is a major limiting factor for chickpea cultivation in all growing regions, causing between 10 and 94% reduction in the potential yield each year [8,9,10,11,12]. For example, in the major growing region India, this disease is estimated to cause a 10% loss of the annual yield [10]. Generally, wilting at early growth stages causes greater loss than that at later stages. A highly susceptible cultivar, under favorable conditions to the pathogen, may wilt within ten days of sowing in a wilt-infested field, whereas tolerant cultivars show general yellowing and drying of the lower leaves with wilting at a later stage, causing less yield damage [11,12,13,14]. These differences in symptoms are also partly due to distinct Fusarium races, of which at least eight have been characterized [11,12,13,14]. Each race is specific to chickpeas and is predominant in different geographic regions.

The targeted agronomic practices may help to prevent the spreading of the disease, such as early sowing and application of fungicides [8,13]. Disease incidence may be further improved with biocontrol agents such as competing non-pathogenic Fusarium races, *Bacillus* sp. or *Pseudomonas fluorescens* [15]. However, integrated disease management that includes the use of genetically resistant varieties is the most viable method of control, especially in chickpea-growing regions with limited resources [9,13,14]. Therefore, developing resistance varieties would be a promising method to overcome the Fusarium wilt problem.

Surveys of chickpea germplasm have identified several resistant lines that have been used to introduce resistant alleles into chickpea varieties [11,12,13,16,17]. Resistance can be monogenic or oligogenic, and it can be race-specific or apply to multiple Fusarium races [10,11,12,13,18,19]. Limited genetic diversity can be a problem as resistance genes to most races occur in one of two genomic regions on chromosome 2 or chromosome 5 [11,12,13,14]. Screening for the resistance sources in natural populations would help to identify new sources of genetic resistance to the disease [12,13,20].

The use of DNA marker technology in breeding programs can make the breeding process quicker and more efficient as new resistance genes can be tagged and eventually identified using linkage and association mapping approaches for populations showing heritable variance in resistance [12,20]. Some DNA markers linked to genes controlling resistance to the disease have been developed using quantitative trait locus (QTL) analysis in mapping populations [12,20,21,22,23,24,25].

In this study, potential novel variations for chickpea Fusarium race 2 resistance were explored using a recombinant inbred line (RIL) family derived from a wide cross between the *C. arietinum* cultivated variety Gokce and the *C. reticulatum* wild accession Kayat-077 that had previously been observed by the authors to show resistance to the locally predominant Fusarium race 2 present in southern Türkiye. RILs were grown alongside parents and check lines in infectious conditions and disease progression was monitored and scored. Inheritance of segregating Fusarium resistance was analyzed. The parents and RILS were genotyped for single nucleotide polymorphism (SNP) markers that were mapped to the CDC Frontier variety reference genome (www.pulseDB.org, accessed on 14 April 2024) and the data were analyzed for the presence of resistance QTLs. The genomic region underlying a significant QTL was interrogated for the presence of potential candidate resistance genes.

## 2. Materials and Methods

### 2.1. Plant and Pathogen Materials

As part of previous research efforts, nested association mapping (NAM) populations that represent valuable resources for chickpea genetics and breeding have been developed [26]. The NAM design simultaneously exploits the advantages of both linkage analysis and association mapping that allow the genetic basis of many complex quantitative traits to be investigated. To develop NAM populations, a cultivated variety of *C. arietinum*, Gokce, was crossed as a female parent to multiple wild Cicer accessions of *C. reticulatum* and *C. echinospermum* species described in [27] as male parents. Each F1 was grown in a glasshouse to obtain approximately 200 F2 seeds. The large families of each cross were then allowed to self-pollinate in the field over four to five generations with five seeds retained per line per generation to develop approximately 150 NAM breeding lines per cross, representing different genetic assortments of the original wild and cultivated chickpea genomes. This study tested the Gocke × Kayat-077 family because this *C. reticulatum* parent had been observed by the authors to show resistance to Fusarium wilt during NAM development compared to the susceptible *C. arietinum* Gokce parent. In addition to the parental lines, resistant check WR315 [10] and susceptible check Besev-079 [27] were grown alongside lines to aid the measurement of Fusarium symptoms.

Cultures of *F. oxysporum* f. sp *ciceris* race 2 were donated by Prof Canan Can, Gaziantep University, Türkiye, and grown in potato dextrose agar (PDA, TM Media, Delhi, India) 39 g/L at 24 ± 2 °C for 10 to 14 days. To obtain spore suspensions, 50 mL of sterile distilled water was poured on the fungus cultures, and the mycelium was scraped with a spatula to transfer the spores to the water. Mycelium residues and agar pieces were removed from the spore suspension using a sterile cheesecloth. The spores in the prepared suspension were counted on hemocytometer Thoma slides (Hawksley & Sons, Lancing, UK), and the suspension concentration was adjusted to 1 × 10^6^ conidias/mL [19].

### 2.2. Greenhouse Experiment

A total of 152 Gokce × Kayat-077 F2:4 lines were grown under typical growing conditions at 25 ± 2 °C and 50–70% humidity in air-conditioned rooms with irrigation as necessary in the greenhouse of Harran University, Türkiye, (37.10 N 39.06 E, 550 m altitude, “hot dry summer” CSA Köppen climate type) during June 2020 and the experiment was repeated under the same conditions with a subset of 83 F3 lines in June 2021. Six seeds from each line were randomly chosen and nicked by a nail clipper to promote germination and planted in a 2:1 mixture of turf compost and perlite (Almar, Darica, Türkiye) in 8 × 6 cm trays. The bench was divided into four blocks of an equal number of lines, each with repeated check lines in each block in an incomplete block augmented design [28].

**Figure 1:**
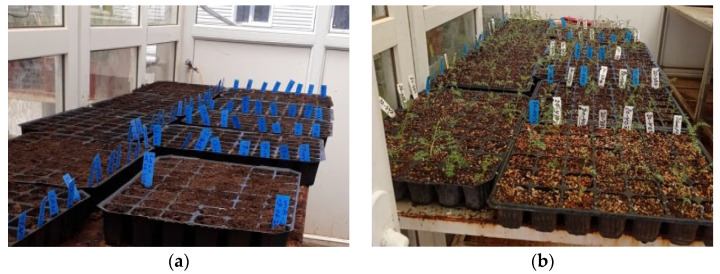

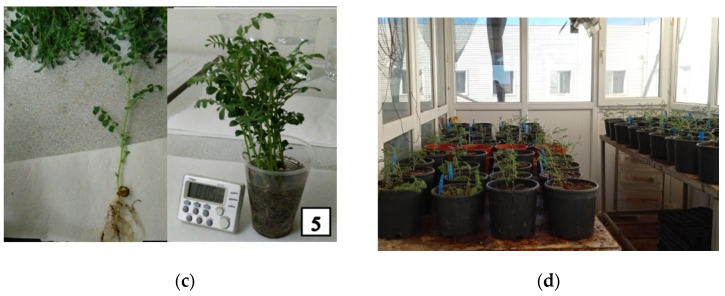
The stages of artificial infection of chickpea with Fusarium in the greenhouse. (**a**) Sowing; (**b**) Seedling stage; (**c**) Fusarium artificial infection of seedlings; (**d**) Replanting of seedlings into pots.

**Figure 2:**
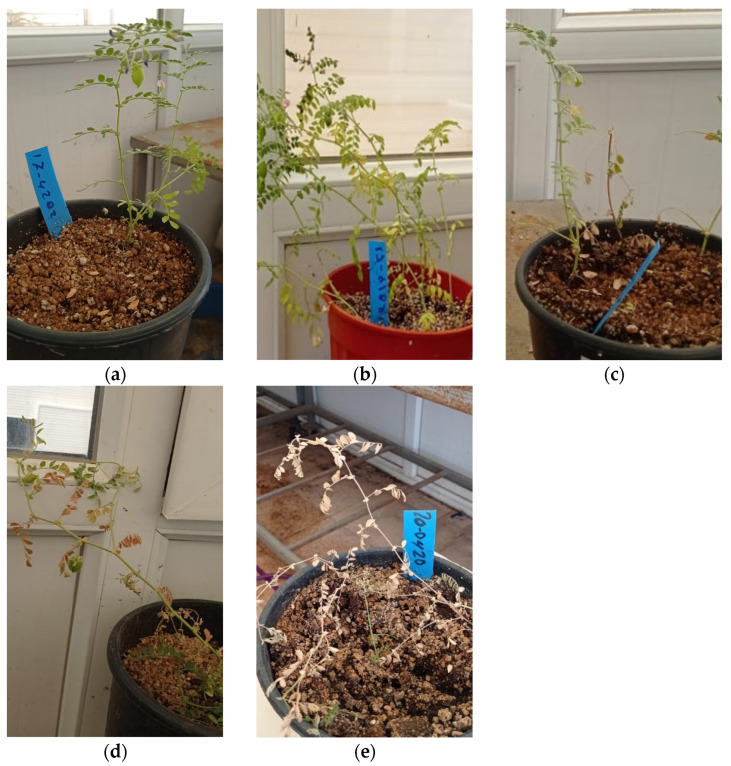
Fusarium disease symptoms based on a 0–4 scale. (**a**) 0: no visible infection; (**b**) 1: slight infection with one or two yellow leaves, which is about 25% of full scale; (**c**) 2: Moderate infection with two or three yellow leaves and about 50% of wilted leaves; (**d**) 3: Extensive infection with almost all yellow leaves and about 75% of wilted leaves and growth inhibited; (**e**) Complete infection with all yellow leaves and 100% wilting and the plant has died.

A Fusarium artificial infection treatment was applied after one month when plants had grown 4–5 nodes with leaves. A pathogenicity test was applied according to the root dipping method [19]. This technique is useful for the evaluation of resistance as it standardizes conditions, ensuring that all test plants are inoculated at the same stage with a constant inoculum load. Chickpea seedlings were removed from the trays without damaging the roots and cleaned from the soil by washing in tap water and the root tips were cut 2–3 cm above the ends with the help of sterile scissors. Then, these roots were dipped in the prepared spore suspension and kept for 5 min. After inoculation, chickpea seedlings were transferred to pots containing compost, with six plants per line in each pot (Figure 1). The injury to roots prior to inoculation ensures that all inoculated plants have a nearly equal chance of infection [19]. Negative control of parents was also applied so that their roots were treated only with water. Disease scales of each line were recorded weekly from 14 days to 49 days after sowing until susceptible lines died based on a 0 to 4 scale [18,29], as described in Figure 2.

### 2.3. Phenotypic Data Analysis

The analyses were performed with Microsoft Excel (Redmond, DC, USA), SPSS v23 (IBM, Armonk, NY, USA), and R v4.1.3 software (https://www.R-project.org, accessed on 28 April 2022). Disease severity was calculated to summarize the disease scale phenotypes of the chickpea lines and checks during the five weeks following inoculation according to Formula (1):

Disease severity = (Sum of all disease ratings × 100)/(Total no. of ratings × maximum disease grade).(1)

Disease development of individual lines over time was also evaluated using area under disease progress curve (AUDPC) scores [30] calculated using the “Agricolae” v1.3-5 R package (https://CRAN.Rproject.org/package=agricolae, accessed on 6 July 2022). Due to the limited number of seeds for each of the RILs, it was necessary to use a single replication of each line in an augmented experimental plot design [28] with four checks that were tested in each of the four blocks. Disease scores, severity, and AUDPC were corrected for block effects using the “plantbreeding” v1.1.1 R package (http://plantbreeding.r-forge.r-project.org, accessed on 6 July 2022). Summary data were calculated for mean disease score progression, severity, and AUDPC frequency distributions.

In order to investigate possible simple Mendelian inheritance mechanisms for AUDPC and disease severity measures, the test lines were classified as resistant, intermediate, or susceptible based on the least significant difference (LSD; α = 0.05) estimated by ANOVA and LSD.test of “Agricolae”. Lines with measures less than the resistant parent plus the LSD were considered resistant, while lines with measures greater than the susceptible parent minus the LSD were considered susceptible genotypes. Lines with intermediate scales were classified as intermediate [31]. The goodness-of-fit to expected segregation ratios of susceptible and resistant lines in RILs for inheritance models for one, two, and three genes were determined by chi-square tests (α = 0.05) [32].

### 2.4. Genotyping and Quantitative Trait Locus (QTL) Analysis

Dried leaf samples from the 2020 growing season were sent to Biosearch Technologies (Hoddesdon, UK) for DNA extraction and genotyping of single nucleotide polymorphisms using the kompetitive allele-specific polymorphism (KASP) approach. The 48 SNPs presented in Table S1 are a subset of 60 SNPs in total that had previously been de, veloped for QTL mapping of interspecific chickpea crosses [26,33] and represent all eight chickpea chromosomes with an average physical map distance of 7.17 Mbp. Raw fluorescence data were converted to SNP genotype calls by setting per SNP fluorescence thresholds to divide the trimodal data distributions representing each parental homozygote and heterozygote, as described in [26]. A genetic map using physical map positions in 1 Mbp units was input into the R package qtl2 v0.30 (https://cran.r-project.org/web/packages/qtl2/index.html, accessed on 21 January 2023), along with genotype and phenotype data for each of the 2020 and 2021 growing seasons, and QTL analysis was performed using the kinship leave one chromosome out (LOCO) option as described in [26]. Significant QTL peaks were identified as regions with logarithm of odds (LOD) scores greater than the 0.95 LOD score quantile estimated from 5000 permutation analyses of the data. The SNP effect sizes of significant QTL peaks were extracted, and the percentage variance explained was calculated according to Formula (2):

PVE = 1 − 10^(−2 LOD/n)^, (2)

where n is the number of measured phenotypes.

### 2.5. Candidate Gene Identification

The genes present in a 1.2 Mbp genomic region centered on significant QTL peak locations and spanning an additional 100 kbp beyond these peaks in either direction of the annotated reference genome CDC Frontier v1.0 were explored using PulseDB tools (https://www.pulsedb.org, accessed on 14 April 2024). This size of region was chosen as it corresponded to the average scale of linkage disequilibrium found in genome resequencing studies of chickpea [34]. For each gene found, the closest matching protein homolog and its description were extracted from the UniProt database, and the top gene ontology term was extracted from the InterPro database.

## 3. Results

### 3.1. Disease Phenotypes

**Figure 3:**
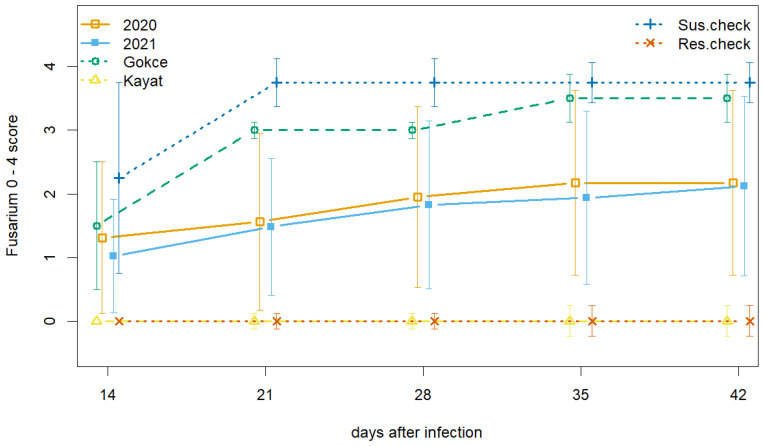
Changes in disease score over five weeks for chickpea Gokce × Kayat family, parental lines, susceptible, and resistant checks inoculated with Fusarium race 2 during the 2020 and 2021 growing seasons. Vertical bars represent standard deviation.

**Figure 4:**
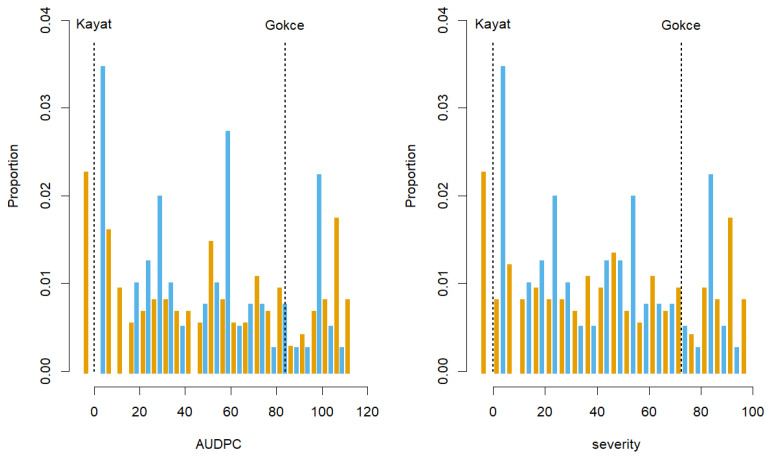
Area under the disease progression curve (AUDPC) and disease severity scores for chickpea Gokce × Kayat family and parental lines five weeks after inoculation with Fusarium race 2 during the 2020 and 2021 growing seasons. Vertical orange and sky blue bars are the proportions of family individuals for the 2020 and 2021 experiments, respectively. Vertical dotted lines are the mean parental values for the 2020 experiment.

The parental and check lines showed widely differing disease symptoms consistent with expectations (Figure 3, Tables S2 and S3). The Kayat-077 parent and resistant check were resistant to Fusarium wilt race 2 with zero disease symptoms in both years. Wilt symptoms developed early in response to race 2 on the Gokce parent and the susceptible check, usually leading to plant death by the end of the five-week monitoring period. The Gokce × Kayat lines showed a wide range of disease symptoms spanning from resistant to susceptible with average scores intermediate between the parents and check lines (Figure 3). Disease symptoms progressed quickly for susceptible Gokce × Kayat lines, similar to parents and check lines. Final AUDPC and severity scores followed a similar pattern with a wide range of symptoms expressed in Gokce × Kayat lines during both seasons (Figure 4). Classification of family lines according to LSD AUDPC and disease severity scores found slightly more susceptible lines during each growing season (Table 1). However, chi-square tests of gene inheritance found that these ratios were not significantly different from a one-to-one ratio indicative of single gene inheritance.

### 3.2. Disease QTLs

Genotype scores are presented in Table S4. The two parents, Gokce and Kayat, and a total of 159 Gokce × Kayat F3 family lines were genotyped, 127 lines from 2020 and 81 from 2021. All SNP markers except one (Ca1C17042, chr6, 3.38 Mbp) produced scorable KASP results. The percent genotype coverage of the 47 scored markers was 95.6% with 43.3% homozygous for Gokce alleles, 35.6% homozygous for Kayat alleles, and 21.1% heterozygous for both parental alleles. One marker (Ca_TOG922889, chr4, 40.59 Mbp) was fixed for Gokce alleles, while two markers (Ca_TOG915738, chr1, 0.32 Mbp and Ca_TOG898271, chr8, 11.63 Mbp) were almost fixed for Kayat alleles.

**Figure 5:**
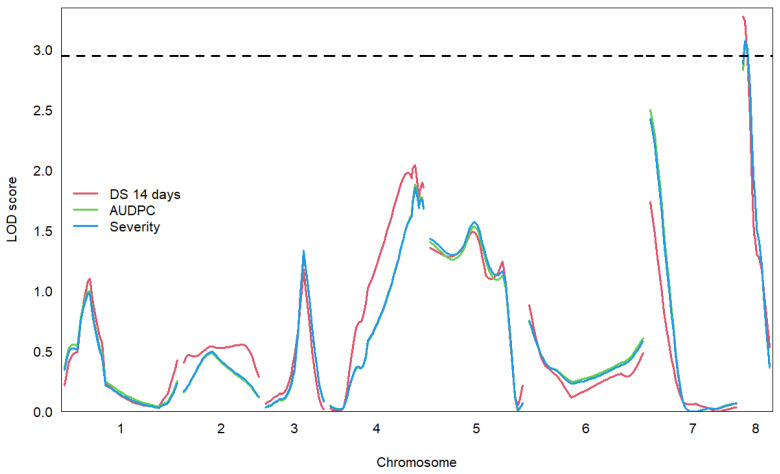
Logarithm of odds (LOD) scores mapped against genome position for Fusarium race 2 disease symptoms in the 2020 Gokce × Kayat family. The horizontal dashed line is the 95% quantile LOD threshold score calculated from 5000 permutations of the data, above which LOD scores indicate significant quantitative trait locus (QTL) positions.

One significant QTL each was identified at a 95% confidence level for disease scores after 14 days, AUDPC, and severity for the 2020 experiment (Table 2, Figure 5). The significant QTLs for each trait were all located at the start of chromosome 8 with a peak LOD score between 0.26 and 1.26 Mbp. The percentage variance explained ranged from 10.41% to 11.21%. No significant QTLs were detected for the 2021 data analysis.

### 3.3. Candidate Disease Resistance Genes

A total of 138 gene models were found in the 1.2 Mbp genomic region surrounding the QTL LOD peaks when this region was compared to the annotated chickpea CDC Frontier v1.0 genome (Table S5). A total of 130 of these gene models were linked to a homolog in the UniProt database and had associated InterPro gene ontology. The homologs of three of these genes were associated with disease-resistance functions (Table 3). The gene annotated Ca_15073 was homologous to *Constitutive Disease Resistance 1* (*CDR1*) of *Arabidopsis thaliana*, with an extracellular aspartic proteinase function that is induced in response to pathogen attack [35]. The genes Ca_15055 and Ca_15063 are homologous to *A. thaliana Ethylene Responsive Transcription Factors 20* and *21* (*ERF20* and *ERF21*) and contain sequence domains that bind to the GCC-box pathogenesis-related promotor element to activate gene expression and transduce stress signals (www.uniprot.org, accessed on 14 April 2024).

## 4. Discussion

Chickpea wilt caused by *F. oxysporum* f. sp. *ciceris* is one of the major yield-limiting factors in chickpea. The main aim of this study was to determine the inheritance of resistance to Fusarium wilt race 2 in a chickpea NAM family derived from a wide cross between a crop variety and its wild crop relative (Gokce × Kayat-077). Our study found evidence that the inheritance of resistance to Fusarium wilt race 2 in this family was controlled by a single QTL.

These results agree with other studies of the genetics of chickpea Fusarium wilt resistance that found it was inherited by a single gene [19,36], which is similar to the monogenic inheritance of resistance to Fusarium races 0, 1A, 3, 4, and 5 that has been observed in other chickpea families [31,37,38,39]. However, other studies have identified segregation for two to three genes conferring resistance to Fusarium race 2 [10,11,12,13,18]. The nature of the resistant allele for each gene in these studies differs in terms of its dominance of expression and impact on slow or late wilting stage, with the presence of resistant alleles at multiple genes conferring complete resistance [10,11,12,13,18,40]. The wilting over time results for this Gokce × Kayat family would appear to be consistent with the typical fast wilting phenotype with a rapid progression of disease symptoms during the first three weeks following infection, leading to plant mortality [10,11,12,13]. However, the causes of the discrepancies among these studies could also be due to different strains of race 2 being used or different environmental or inoculation conditions.

This study found evidence of a potential QTL for chickpea Fusarium wilt resistance on chromosome 8. This QTL was only found for some disease measures during the 2020 growing season, although a supporting inheritance pattern was found in the 2021 growing season data. The lack of significant QTLs during the 2021 growing season might have been due to a smaller sample size (81 in 2021 compared to 127 in 2020) and/or using genotypes for the 2020 F2:4 generation to map the 2021 F3 generation. The relatively simple inheritance of chickpea Fusarium wilt resistance is supported by other QTL mapping studies that have identified a few genomic locations that host most resistance genes [12]. Two QTL hotspots for resistant genes to multiple Fusarium races have been mapped to chickpea chromosome 2 [11,12,13], while another QTL conferring resistance to race 0 has been mapped to chromosome 5 [11,13,22]. Patterns of inheritance in other studies suggest that resistance alleles of additional genes at other unlinked and as yet unmapped genomic locations are required to confer full resistance, highlighting the value of finding new loci that contribute to chickpea Fusarium wilt resistance [12,13,41,42]. The chickpea Fusarium wilt resistance QTL reported in this paper could constitute one such new locus for investigation.

Public genomic resources for chickpea allow QTLs to be screened for potential candidate genes for follow-up studies [43]. Screening of the potential chickpea Fusarium wilt resistance QTL identified in this study suggested three candidate genes. Two of these genes, Ca_15055 and Ca_15063, are homologs of *A. thaliana ERF20* and *ERF21* genes that transduce various stress stimuli into appropriate plant responses. These genes also contain a GCC-box pathogenesis-related promotor element, making them potential candidates for chickpea Fusarium resistance also [44,45,46]. The third gene, Ca_15073, is homologous to the Arabidopsis *CDR1* gene, an extracellular aspartic proteinase that has been implicated in pathogen resistance by possibly triggering a mobile protein systemic acquired resistance elicitor from the site of attack [35,47]. Aspartic proteases have been implicated in other plant–pathogen interactions with other potential modes of action, such as specific cleavage of pathogen effector proteins and antimicrobial activity [47,48,49,50]. These findings might suggest new research directions to investigate the molecular mechanisms of chickpea Fusarium wilt resistance. These downstream mechanisms are still being elucidated through transcriptomics and metabolomics approaches, implicating the expression of antifungal metabolites, reactive oxidative species, cell-wall remodeling, and the salicylic acid signaling pathway [12,13,51,52,53,54], which can also be race-specific [55].

## 5. Conclusions

This study supports the value of investigating natural genetic variation in new accessions of crop wild relatives to discover more about disease resistance. Fusarium wilt race 2 inoculation tests of a cultivated chickpea × wild *C. reticulatum* accession identified a potential QTL for resistance in a distinct genomic region to previous studies and some candidate genes to follow up in future studies. In addition to potential practical implications to efforts to breed for improved chickpea resistance to Fusarium wilt, a major yield-limiting factor for chickpea worldwide, these results also contribute to our understanding of the complex molecular responses involved in this host–pathogen interaction.

## Figures and Tables

**Table 1:** Classification of chickpea Golce × Kayat family lines as susceptible or resistant to Fusarium race 2 based on parental least square differences and chi-square tests of gene inheritance during the 2020 and 2021 growing seasons. Inheritance ratios with greater than a 0.05 probability are shown in bold text.

Year	Measure	Susceptible Count	Resistant Count	Inheritance Ratio Tested	Chi-Square Statistic
2020	AUDPC	39	29	1:1	**1.47**
				3:1	11.29
				7:1	56.50
2020	Severity	42	30	1:1	**2.00**
				3:1	10.67
				7:1	56.00
2021	AUDPC	18	15	1:1	**0.27**
				3:1	7.36
				7:1	43.17
2021	Severity	24	15	1:1	**2.08**
				3:1	3.77
				7:1	24.03

**Table 2:** Summary quantitative trait locus analysis results for Fusarium disease traits of a chickpea Gokce × Kayat F2:4 mapping family. Locations list the chromosome number, peak logarithm of odds (LOD) score position in Mbp, and 1.5 LOD confidence limits in parentheses. LOD is a peak LOD score. PVE is the percentage variance explained. SNP is a single nucleotide polymorphism marker name with up to two adjacent SNPs listed. Mu is the mean effect size. RR is the effect size of the Gokce reference allele homozygote. RO is the effect size of the heterozygous genotype. OO is the effect size of the Kayat other allele. Add. effect is the additive effect size.

Trait	Disease Score 7 Days	AUDPC	Severity
Location	8, 0.26 (0.26–5.05)	8, 1.26 (0.26–11.63)	8, 1.26 (0.26–6.03)
LOD	3.28	3.03	3.07
PVE	11.21	10.41	10.53
SNP	Ca1C23895	Ca1C23895, Ca_TOG898231	Ca1C23895, Ca_TOG898231
Mu	1.48	57.34	50.52
RR	−0.34	−9.66	−8.48
RO	0.79	25.52	22.63
OO	−0.45	−15.855	−14.15
Add. effect	−0.05	−3.10	−2.83

**Table 3:** Candidate disease resistance genes within a 1.2 Mkp genomic region of the chickpea CDC Frontier rv1.0 reference genome associated with a quantitative trait locus (QTL) for Fusarium race 2 disease symptoms in a Gokce × Kayat mapping family. Gene names and genomic locations follow the conventions of PulseDB (www.pulsedb.org, accessed on 14 April 2024).

Gene Name	Location	Uniprot Homology	InterPro Annotation
*Ca_15055*	Ca8: 1235430 … 1235960	*ERF20_ARATH*	IPR001471, Ethylene-responsive transcription factor *ERF020*
*Ca_15063*	Ca8: 1169327 … 1169752	*ERF21_ARATH*	IPR001471, Ethylene-responsive transcription factor *ERF021*
*Ca_15073*	Ca8: 1093219 … 1094244	*CDR1_ARATH*	IPR001461, Aspartic proteinase *CDR1*

## Data Availability

The original contributions presented in the study are included in the article/Supplementary Materials; further inquiries can be directed to the corresponding author.

## Supplementary Materials

The following supporting information can be downloaded at: https://www.mdpi.com/article/10.3390/genes15060819/s1, Table S1: Genotyped single nucleotide polymorphism (SNP) marker information. SNP names match those used for kompetitive allele-specific polymorphism (KASP) genotyping at Bioscience Technologies. Table S2: Chickpea Fusarium wilt disease scores, area under disease curve, and severity for the 2020 glasshouse experiment of the cultivated Gokce × wild *C. reticulatum* Kayat-077 F2:4 recombinant inbred line (RIL) family. Column 1 is the mapping family code. Columns 2, 3, and 4 are the individual identifiers for genotyping, the 2020 and the 2021 glasshouse experiments. Column 5 is the glasshouse block number. Columns FUS1 to FUS5 are weekly disease scores from 14 to 49 days, respectively. Columns FUS1mod to FUS5mod are augmented design corrected weekly disease scores. AUDPC area under disease progress curve values. Severity is disease severity values. Table S3: Chickpea Fusarium wilt disease scores, area under disease curve, and severity for the 2021 glasshouse experiment of the cultivated Gokce × wild *C. reticulatum* Kayat-077 F3 RIL family. Columns 1, 2, and 3 are the mapping of individual identifiers for genotyping, the 2020 and the 2021 glasshouse experiments. Columns FUS1 to FUS5 are weekly disease scores from 14 to 49 days, respectively. AUDPC area under disease progress curve values. Severity is disease severity values. Table S4: SNP genotypes for the cultivated Gokce × wild *C. reticulatum* Kayat-077 F3 RIL family. Column 1 is the individual identifiers. Columns 2 to end are the genotype scores for each SNP ordered according to genomic position. R (reference) is the cultivated Gokce parental allele. O (other) is the wild *C. reticulatum* Kayat-077 parental allele.—indicates unscored genotypes. Table S5: Gene models, UniProt gene homologs, and InterPro gene ontologies, present within the 1.2 Mbp region surrounding the quantitative trait locus (QTL) for Chickpea Fusarium wilt resistance according to the Chickpea CDC Frontier reference genome v1.0.
